# Bioconversion of *Chamaecyparis obtusa* Leaves with *Phellinus linteus* Mycelium Modulates Antioxidant and Anti-Inflammatory Activities

**DOI:** 10.3390/cimb48010026

**Published:** 2025-12-25

**Authors:** Soo Ah Jeong, Abdullah Talukder, Yeong Hwan Jeong, Myeong Gwan Son, Gi Hyeon Kim, Beong Ou Lim

**Affiliations:** 1 BK21 Program, Department of Medicinal Biosciences, Graduate School, Konkuk University, Chungju 27478, Chungcheongbuk-do, Republic of Korea; jje318@kku.ac.kr (S.A.J.); mdtoabdullah@gmail.com (A.T.); mijo6064@kku.ac.kr (Y.H.J.); krutoc@kku.ac.kr (M.G.S.); rlgusfldj@kku.ac.kr (G.H.K.); 2 Human Bioscience Corp., 268 Chungwondaero, Chungju 27478, Chungcheongbuk-do, Republic of Korea

**Keywords:** *Chamaecyparis obtusa*, *Phellinus linteus*, antioxidant, anti-inflammatory, cytotoxicity, bioconversion, macrophages

## Abstract

*Chamaecyparis obtusa* (Hinoki cypress) has been extensively studied for its leaves and bark, which are known to contain high levels of polyphenols and flavonoids with potent bioactivities. In this study, the phytochemical transformation and changes in bioactivity of *C. obtusa* leaves were investigated through bioconversion using the mycelium of *Phellinus linteus*. Initially, extracts of *C. obtusa* leaves were prepared using distilled water (COD) and 99% ethanol (COE), and the bioconverted extract, CPE-1. The extracts were evaluated for antioxidant potential (DPPH and ABTS radical scavenging), cytotoxicity, antibacterial efficacy, and anti-inflammatory activity in LPS-stimulated RAW 264.7 macrophages. The results indicated that CPE-1 exhibited the highest overall efficacy among the tested extracts, showing antioxidant activity comparable to that of BHT and ascorbic acid, while presenting relatively lower antimicrobial effects against *Staphylococcus aureus* and *Bacillus* spp. However, in an in vitro cellular model, CPE-1 significantly enhanced anti-inflammatory effects, including notable inhibition of nitric oxide (NO) production, suppression of COX-2 and iNOS expression, and inhibition of ERK and JNK phosphorylation. Its antioxidant activity remained strong, exhibiting radical scavenging capabilities comparable to those of synthetic controls (BHT and ascorbic acid). HPLC analysis confirmed that bioconversion successfully modified the phytochemical profile of *C. obtusa*, yielding metabolites with enhanced potency while preserving stable, beneficial compounds like gallic acid. These findings collectively establish fungal biotransformation as an effective technology for upgrading plant-derived extracts into potent, multifunctional bioactive materials suitable for therapeutic or functional food applications.

## 1. Introduction

Inflammation is a fundamental biological response of the human body to harmful stimuli, including pathogens, damaged cells, and toxic compounds. While a properly regulated inflammatory response is protective and essential for healing, excessive or chronic inflammation can damage healthy tissue and contribute to the development of diseases such as cancer, cardiovascular disorders, and metabolic syndromes [1].

Key proinflammatory mediators include cytokines such as tumour necrosis factor-alpha (TNF-α), interleukin-1 beta (IL-1β), interleukin-6 (IL-6), lipid-derived molecules like prostaglandins, and reactive oxygen species (ROS) [2]. Acute inflammation is typically a short-lived immune response to infections or harmful agents, as supported by findings from the University of Florida College of Public Health and Health Professions [3]. In contrast, chronic inflammation may persist for months or years and is implicated in the pathogenesis of several chronic conditions, including kidney disease, cardiovascular disease, cancer, and type 2 diabetes. A recent study led by Mainous et al. reported that systemic inflammation affects approximately 34.6% of American adults [4].

Macrophages are crucial immune cells involved in detecting, engulfing, and destroying pathogens and apoptotic cells [5]. Lipopolysaccharide (LPS), a component of the outer membrane of Gram-negative bacteria, activates macrophages through Toll-like receptor 4 (TLR4), initiating signalling pathways such as NF-κB and MAPK via the adaptor protein MYD88 [6,7]. This leads to the production of inflammatory mediators, including nitric oxide (NO) and prostaglandin E2 (PGE2), upregulation of inducible nitric oxide synthase (iNOS) and cyclooxygenase-2 (COX-2), and the release of cytokines such as IL-1β, IL-6, and TNF-α [8,9,10]. Prolonged or excessive activation of these pathways can cause tissue damage. Although corticosteroids are commonly used to treat chronic inflammatory skin diseases, long-term use can cause significant side effects [11,12]. Consequently, there is growing interest in identifying natural and safer alternatives for anti-inflammatory therapy.

*Chamaecyparis obtusa* (Hinoki cypress), a member of the Cupressaceae family native to East Asia, has been traditionally used to alleviate allergic and inflammatory symptoms. Modern pharmacological studies have confirmed its antioxidant, antimicrobial, and anti-inflammatory activities, largely attributed to flavonoids, terpenoids, and phenolic compounds [13,14]. The exploration of plant-derived bioactive compounds as therapeutic agents is a rapidly developing area of biomedical research [15,16]. Previous research has demonstrated that extracts from *Chamaecyparis obtusa* leaves (COD) and cypress bark (CBE) exhibit antioxidant, anticancer, and dermatological benefits. These extracts are rich in flavonoids, polyphenols, and other bioactive compounds [2,17]. One study showed that *C. obtusa* leaf extract suppresses the JAK/STAT signalling pathway in RAW264.7 macrophage cells, thereby inhibiting inflammatory responses [18]. Moreover, when fermented with Ganoderma applanatum, the *C. obtusa* leaf extract (70COLGA) significantly inhibited LPS-induced NO production, iNOS, and COX-2 expression in RAW264.7 cells, while reducing the cytotoxicity observed with unfermented extracts [2]. Our previous research also demonstrated that an extract from *C. obtusa* bark suppressed the CREB and MITF signalling pathways in α-MSH-stimulated B16F10 cells, resulting in reduced melanogenesis [17].

In recent years, biotechnological biotransformation has emerged as a promising approach to enhance the pharmacological properties of plant-derived materials. Medicinal fungi such as *Phellinus linteus* possess diverse enzymatic systems capable of converting natural precursors into more bioactive or bioavailable forms [19,20]. This process can increase phenolic content, alter structural diversity, and generate metabolites with improved antioxidant and anti-inflammatory activity [19,20,21]. Such fungal-assisted transformations have attracted attention for producing multifunctional materials suitable for pharmaceutical and functional food applications.

The present study introduces a novel bioconverted extract of *C. obtusa* (CPE-1), produced by fermenting *C. obtusa* leaves with *Phellinus linteus* mycelium. This approach integrates plant fungus co-metabolism to enhance biological efficacy. We compared the antioxidant, antibacterial, and anti-inflammatory activities of ethanolic (COE), aqueous (COD), and bioconverted (CPE-1) extracts and explored the molecular mechanisms underlying their effects by evaluating iNOS, COX-2, and MAPK (ERK and JNK) signalling in LPS-stimulated RAW264.7 macrophages. This study highlights the biotechnological potential of fungal biotransformation as a sustainable method to develop functionally enhanced natural materials for therapeutic and nutraceutical applications.

## 2. Materials and Methods

### 2.1. Chemicals and Materials

Dibutyl hydroxy toluene (BHT), ascorbic acid (AA), Folin–Ciocalteau reagent (FC reagent), gallic acid, sodium nitrite (NaNO_2_), trichloroacetic acid (TCA), 3-(4,5-dimethylthiazol-2-yl)-2,5-diphenyl tetrazolium bromide (MTT), ferric chloride (FeCl_3_) dimethyl sulfoxide (DMSO) 2,2-diphenyl-1-picrylhydrazyl (DPPH), sodium hydroxide (NaOH) ferrous sulphate heptahydrate (FeSO_4_), ammonium thiocyanate, 2′7′-dichlorodihydrofluorescein diacetate (H2DCF-DA), trifluoroacetic acid, potassium persulfate, 2,2′-azinobis-(3-ethylbenzothiazoline-6- sulfonic acid) (ABTS), anhydrous sodium phosphate dibasic, anhydrous sodium phosphate monobasic, and EDTA were purchased from Sigma Chemical Co. (St. Louis, MO, USA). The pBR322 DNA, 6X DNA loading dyes, stripping buffer, PageRuler Prestained Protein Ladder and Syncronis C_18_ Column were purchased from Thermo Scientific (Waltham, MA, USA). From Bio Basic Inc. (Markham, ON, Canada), Agarose A. DPBS, Dulbecco’s Modified Eagle’s Medium (DMEM), Trypsin EDTA, Foetal Bovine Serum (FBS) and Penicillin-streptomycin (PS) were purchased from WELGENE Inc. (Gyeongsan-si, Republic of Korea). Antibodies recognising p-JNK, GAPDH, COX-2, iNOS, and p-ERK were acquired from Cell Signalling Technology (Beverly, MA, USA). All chemicals were used without further purification.

### 2.2. Preparation of Extracts

**Figure 1:**
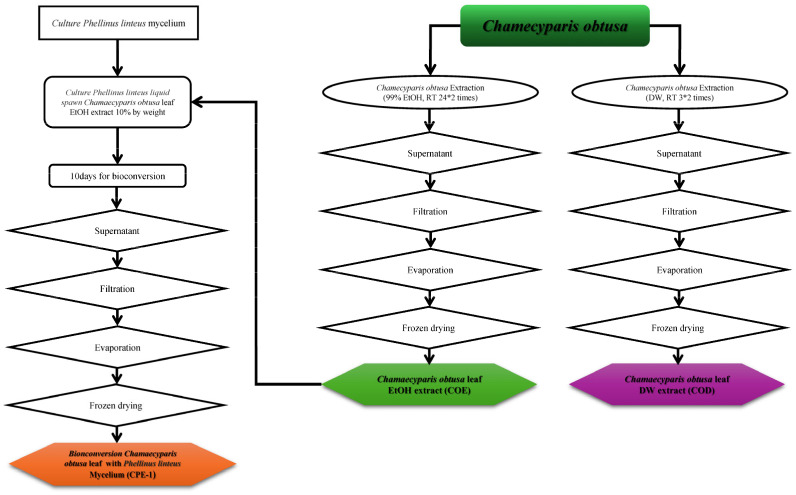
Schematic illustration of the preparation of *Chamaecyparis obtusa* leaf extracts and the bioconverted extract. *C. obtusa* leaves were extracted either with 99% ethanol at room temperature (24 h, two times) to obtain the ethanol extract (COE) or with distilled water under reflux (3 h, three times) to obtain the water extract (COD), followed by filtration, evaporation, and freeze-drying. For bioconversion, *Phellinus linteus* mycelium was cultured in liquid medium supplemented with *C. obtusa* leaf ethanol extract (10% *w*/*w*) for 10 days, after which the supernatant was collected, filtered, evaporated, and freeze-dried to yield the bioconverted extract (CPE-1).

In the beginning, two solvents were used to extract the source material as illustrated in Figure 1, *Chamaecyparis obtusa* (*C. obtusa*). The DW extract was first extracted twice at room temperature for three hours. 99% EtOH was used to extract the EtOH extract, and the extraction process was carried out twice over the period of one full day at room temperature. Whatman No. 1 filter paper (GE Healthcare UK Limited, Hertfordshire, UK) was then used to filter both extracts. A vacuum rotary evaporator was used to evaporate this extract.

Finally, after filtering and concentrating the EtOH extract, the new bioconversion material *P. linteus* mycelia were pre-cultured on potato dextrose agar (PDA) at 28 °C for 7 days. Liquid fermentation was carried out in potato dextrose broth (PDB) at 28 °C, 150 rpm, for 10 days. After reaching the active growth phase, the fungal culture was inoculated directly into the extract 10% *v*/*w* mixture for bioconversion. It was then filtered via Whatman No. 6 filter paper (GE Healthcare UK Limited, UK) to concentrate it. After being condensed, each sample was lyophilised at −108 °C.

In the end, until it was used, the extract was kept in a refrigerator at −20 °C.

### 2.3. Determination of Total Phenolic Content

Following a small modification, the Folin–Ciocalteu colourimetric method, such as the FC reagent method [22,23], was used to determine the total phenolic content. The standard curve was developed with gallic acid. The concentration of gallic acid in water was between 0 and 100 mg/mL, and all samples were generated at 10 mg/mL [24]. In each well, 40 μL of each sample (COD, COE, and CPE-1) was mixed three times, followed by 20 μL of 1 M Folin–Ciocalteu reagent (FC reagent) and 60 μL of 20% (*w*/*v*) sodium carbonate (Na_2_CO_3_). For half an hour, the mixtures were kept at room temperature (RT) in the dark. The absorbance was taken at 700 nm with a Versamax microplate reader (Molecular Devices, San Jose, CA, USA). The results were denoted as mg of GAE per 100 g of dry mass from the calibration curve of GA.

### 2.4. Determination of Total Flavonoid Content

The content of total flavonoids was measured as previously mentioned [22,24]. Sample and standard solution concentrations were 10 mg/mL and 0–100 µg/mL, respectively. To summarise it briefly, 125 µL of DW was combined with 25 µL of each sample (COD, COE, or CPE-1) or reference solution, and then 8 µL of a 5% (*w*/*v*) NaNO_2_ solution was added. and reacted for five minutes at room temperature. Following the addition of 15 mL of 10% aluminium chloride and a 6 min reaction with 1 M sodium hydroxide (NaOH) of 50 mL and 27 mL, distilled water was added to the ELISA reader, and the absorbance was measured at 510 nm. A standard calibration curve derived from the catechin standard and represented as catechin equivalents in µg per mg of extract was used to determine the total flavonoid content of each sample. Every sample was examined three times.

### 2.5. DPPH Radical Scavenging Activity

Using a previously published method, the diphenyl-2-picrylhydrazyl free radical-scavenging capabilities of water and 70% (*v*/*v*) ethanol extracts were assessed [25,26]. BHT, ascorbic acid, and different concentrations of the sample (ranging from 0.25 to 4 mg/mL) were synthesised. A 100 mL DPPH solution was combined with 100 mL of each sample/standard solution. The combination was then allowed to incubate at room temperature (RT) for half an hour. Each group solution’s absorbance was measured at 517 nm. The control was made using the same procedure as the sample and standard. Using the following formula, the DPPH radical scavenging activity was determined:

DPPH free radical scavenging activity (%) = {(C − D) − (A − B)}/(C − D) × 100

Meaning of A is absorbance of DPPH + sample/standard, B is absorbance of sample/standard + methanol, C is absorbance of DPPH + DW/methanol, and D is absorbance of methanol + DW.

### 2.6. ABTS Radical Scavenging Activity

The extracts’ ability to scavenge ABTS+ radicals was assessed using partially modified versions of the previously published methods [22,25,27]. In order to create ABTS+, ABTS powder (bought from Sigma Aldrich, St. Louis, MO, USA) was first dissolved in D.W. to a concentration of 7 mM. The mixture was then incubated at room temperature for 14 h before use, resulting in a final concentration of 2.45 mM. Freshly made ABTS solution was diluted with 0.01 M PBS (phosphate-buffered saline, pH 7.4) for each experiment in order to bring its absorbance within 0.70 ± 0.02 at a wavelength of 734 nm. Then 0.1 mL of various concentrations of the samples (COD, COE, CPE-1), BHT(butyl-hydroxytoluene) and AA (Ascrobic acid) in 0.25–4 mg/mL were mixed with 0.9 mL of ABTS solution. The final step is measuring absorbances at 734 nm after incubation at RT for 5 min. The ABTS scavenging activity was calculated using the following equation:

ABTS radical scavenging activity (%) = {(C − D) − (A − B)}/(C − D) × 100

To add an explanation, A = Optical Density of ABTS solution + sample/standard, B = Optical Density of potassium persulfate + sample/standard, C = Optical Density of ABTS solution + DW/methanol, and D = Optical Density of potassium persulfate + DW/methanol.

### 2.7. Determination of Reducing Power

Following small modifications, the ferric reducing power activities of COD, COE, and CPE-1, as well as positive controls such as BHT and AA, were assessed using a previously published technique [22]. 2.5 mL of 0.2 M sodium phosphate buffer (pH 6.6) and 2.5 mL of potassium ferricyanide (10 mg/mL) solution were mixed with 1 mL of each sample or standard reagent in a range of concentrations (0.25–4.00 mg/mL). The mixture was subsequently incorporated with 2.5 mL of TCA (trichloroacetic acid, 100 mg/mL) and centrifuged for 10 min at 3000 rpm after being incubated for 30 min at 50 °C in a water bath. After that, 0.25 mL of DW and 0.25 mL of supernatant were combined, and 0.5 mL of FeCl_3_ (0.1% (*w*/*v*)) was added to the mixture. Higher absorbance indicated greater reducing power. So the Optical Density was measured at 700 nm.

### 2.8. Oxidative DNA Damage Protective Activity

6X loading and plasmid DNA, specifically pBR322, was purchased from Thermo Scientific. According to a previous study, the COD, COE, and CPE-1’s capacity to stop hydrogen peroxide-induced DNA damage was examined [26]. Three millilitres of 30% hydrogen peroxide (H_2_O_2_) (*v*/*v*), five millilitres of D.W., two millilitres of 0.08 mM iron(II) sulphate heptahydrate (FeSO_4_), and two millilitres of both extracts at different concentrations (0.25, 0.5, and 1 mg/mL) were combined with pBR322 (1 µL, 0.5 µg/µL). Two microliters of 6X loading dye were then added to the mixture, and it was incubated for an hour at 37 °C. The combination was electrophoresed using a 0.8% agarose gel (Mupid-2plus; Advance Co., Ltd., Osaka, Japan) at room temperature (100 V). Ethidium bromide (EtBr) was used to stain DNA (deoxyribonucleic acid) bands, including supercoiled, linear, and open circular, and the gels were then scanned using the ChemiDoc MP Imaging System (BIO RAD, Hercules, CA, USA).

### 2.9. Antibacterial Activity

The antibacterial activity of the extracts was determined using the well diffusion method, following standard microbiological procedures. Two standard reference strains (*Staphylococcus aureus*, ATCC; *Bacillus subtilis*, ATCC) were obtained from the institutional microbial culture collection. Nutrient agar (NA) plates were prepared, and 0.1 mL of soft agar containing each bacterial strain (nutrient broth culture) was overlaid onto the NA surface and allowed to solidify. Wells of uniform diameter were created using a sterile pipette tip. The extract samples were dissolved in distilled water (DW), and 0.05 mL of each sample was dispensed into the wells.

For *S. aureus*, plates were incubated under anaerobic conditions at 37 °C for 24–48 h. Antibacterial activity was quantified by measuring the diameter of the inhibition zone (mm) surrounding each well. The assay for *B. subtilis* was conducted using the same procedure and incubation conditions. All experiments were performed in triplicate, and results are presented as mean ± standard deviation (SD).

### 2.10. Cell Viability

Murine macrophage RAW 264.7 cell line was purchased from the Korean Cell Line Bank (Seoul, Republic of Korea) and maintained in DMEM with 10% FBS and 1% P.S in a cell incubator. Cells were cultured in an incubator at 37 °C with 5% CO_2_ and 95% humidified conditions. Cell viability was measured by the MTT assay. The RAW 264.7 cells were seeded in a 96-well plate as 5 × 10^4^ cells/well and incubated for 24 h. After that, Raw 264.7 cells were treated with different concentrations of COD, COE (10–1000) μg/mL, CPE-1 (1–10) μg/mL and Lipopolysaccharide (LPS) 1 μg/mL for more 24 h. After incubation, 0.5 mg/mL MTT (final concentration) was added to every well and kept in the incubator for 2 h. The supernatant was then removed, and DMSO (Dimethyl sulfoxide) was added to solubilise the formazan and stored at room temperature in a dark condition for 10 min and optical density was taken at 570 nm (Versa max, Molecular Devices, CA, USA) and analysed by SoftMax pro 7.1 edition. The control group was considered 100%.

### 2.11. Determination of Nitric Oxide (NO) Production

Nitric oxide (NO) production by LPS-induced RAW 264.7 cells was evaluated by Griess assay [28]. The cells were seeded as 5 × 10^4^ cells/well in a 96-well plate and incubated for 24 h. Afterwards, cells were treated with LPS (1 μg/mL) and COD, COE (10–1000) μg/mL and CPE-1 (1–10) μg/mL for another 18 h, where the control group contained only DMEM. Then, 80 μL supernatant from each well was collected and added to a new 96-well plate and mixed with the same amount of Griess reagent. The mixture was shaken in a shaker at room temperature (RT) under dark conditions for 10 min before taking absorbance at 540 nm. Nitric Oxide (NO) was determined from the standard curve of sodium nitrite (NaNO_2_).

### 2.12. Western Blot Analysis

RAW 264.7 cells in DMEM were seeded as 1.5 × 10^6^ cells/well a 100 mm dish were incubated for 24 h at 37 °C in a CO_2_ incubator. Then, COD and COE (10, 25, 50, 100) μg/mL were treated, respectively, and cells were treated with LPS (1 μg/mL), and then cultured for 24 h. In the case of CPE-1, (1, 2.5, 5) μg/mL was treated, and the experiment was carried out. Next, total protein was extracted using PRO-PREP buffer purchased by iNtRON Biotechnology and the protein was separated from the cell lysate by centrifugation at 14,000 rpm at 4 °C [29,30]. An equal amount of protein (20 μg/lane) was subjected to a 12% SDS polyacrylamide gel. After electrophoresis, protein samples were transferred into a PVDF (Polyvinylidene fluoride) membrane and blocked with 5% Bovine Serum Albumin (BSA). The membranes were incubated with corresponding primary antibodies at 4 °C over-night, then the membranes were incubated with secondary antibody(anti-rabbit) at room temperature for 1 h. Then, the immunosignals in membranes were visualised by ECL kit (enhanced chemiluminescence) in ChemiDoc MP Imaging System (BIO RAD). The band intensity was quantified by ImageJ software (version 1.54).

### 2.13. Determination of Cell Intracellular ROS Using Flow Cytometry

Intracellular ROS (reactive oxygen species) levels were measured by detecting the fluorescent intensity of cells as described [31]. RAW 264.7 cells were seeded at a density of 1 × 10^6^ cells/well into 6-well plates were pre-treated with various concentrations (1, 2.5, and 5 μg/mL) of CPE-1 and NAC (N-acetyl-L-cysteine, 20 mM) for 1 h. Then the cells were stimulated with medium containing LPS (100 ng/mL) at 37 °C for 30 min. Cells were washed with PBS (Phosphate-buffered saline) and incubated at 37 °C for 30 min in dark conditions with the probe at a final concentration of 10 µM DCF-DA. Cells were washed with cold phosphate-buffered saline and gently scraped. The fluorescent intensity was analysed at an excitation wavelength of 485 nm and an emission wavelength of 535 nm using a FACS Calibur flow cytometer (Becton & Dickinson Co., Franklin Lakes, NJ, USA).

### 2.14. HPLC Analysis

Bioactive compounds of COE and CPE-1 were determined in HPLC (Thermo Scientific Dionex Ultimate 3000 series) consisted of a C_18_ analytical column (Thermo Fisher Scientific, Waltham, MA, USA) that 4.6 × 250 mm, 5 μm particle size, Supelco C_18_ column. For identifying bioactive compounds in COE and CPE-1, 0.1% Trifluoroacetic acid (TFA) in water was used as mobile phase A and 0.1% TFA in acetonitrile was used as mobile phase B (elution conditions: 0 min, 60% B; 30 min, 90% B; 40 min, 90% B; flow rate, 0.6 mL/min; injection volume, 20 μL; wavelength, 275 nm and column temperature 28 °C). And samples were dissolved in methanol (MeOH) and filtered through a 0.45 μm membrane filter. All solvents and mobile phases were HPLC grade, and polyphenols were confirmed by matching the retention time with the standards.

### 2.15. Statistical Analysis

The results were demonstrated as mean ± SD for all experimental data. Data analyses were performed with Microsoft Excel Office 365 edition and GraphPad Prism 5.0 software (GraphPad Software, Inc., San Diego, CA, USA) using one-way analysis of variance according to Tukey’s Multiple Comparison Test. A *p*-value less than 0.05 was considered statistically significant. All experimental data were performed at least in triplicate.

## 3. Results

### 3.1. Total Phenolic and Flavonoid Contents

The leaves, roots, and flowers of many plants contain phenolic and flavonoid chemicals. Most commonly found in plants, phenols are biofunctional secondary metabolites with anti-inflammatory and free radical-scavenging properties. One of the main impacts of phenolics in the mammalian body is antioxidant activity [32]. The total phenolic content in COD was 366.246 ± 8.76 µg GAE, COE were 177.1692 ± 7.899 µg GAE, and CPE-1 was 62.90404 ± 3.999907 µg GAE per mg of extract, each. On the other hand, the total flavonoid content in COD was 26.751 ± 1.31 µg CE, COE were 109.6914 ± 2.823 µg CE, and CPE-1 was 137.8148 ± 3.591972 µg CE per mg of extract, respectively. These results are shown in Table 1 below. All results, each of the total phenolic and flavonoid contents were expressed in gallic acid equivalents (GAE) and catechin equivalents (CE), and calculated from the standard calibration curve. This result shows that the content of flavonoids increased in CPE-1 than in COD and COE, which are raw materials.

### 3.2. Evaluating the Antioxidant and Cytotoxic Potential of COD, COE, and CPE-1 Extracts

**Figure 2:**
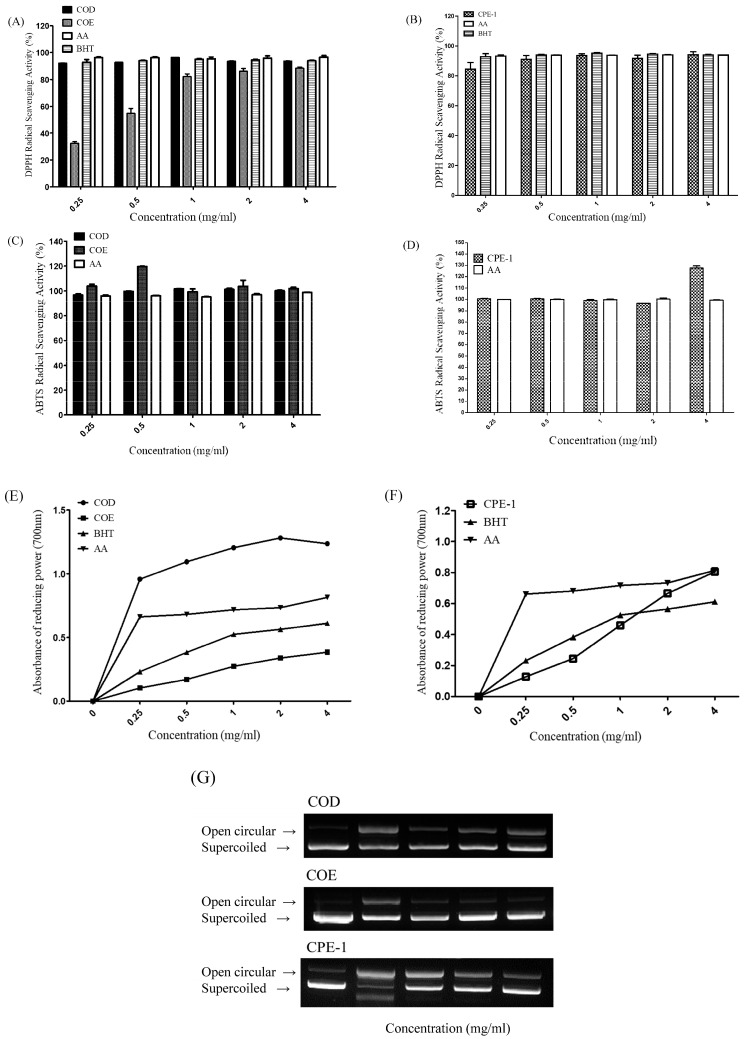

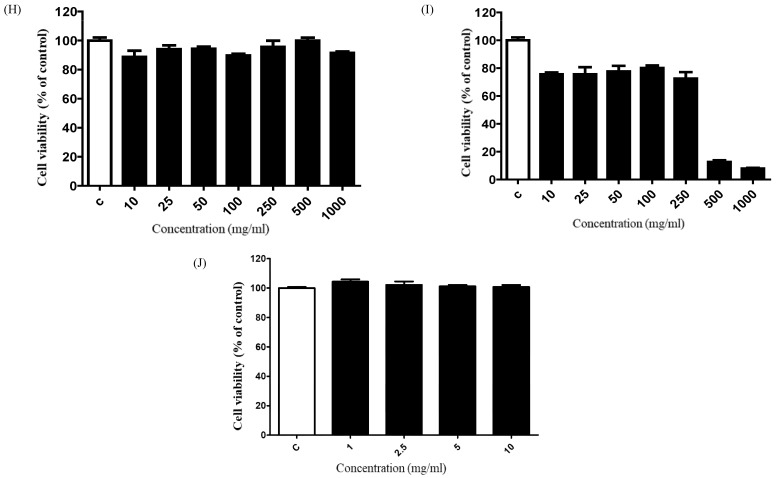
Evaluation of the Antioxidant and Cytotoxic Activities of COD, COE, and CPE-1 Extracts. (**A**–**D**) DPPH and ABTS radical scavenging activities of COD, COE, and CPE-1 extracts compared to standard antioxidants ascorbic acid (AA) and butylated hydroxytoluene (BHT) at various concentrations (0.25–5 mg/mL). Evaluation of the Antioxidant and Cytotoxic Activities of COD, COE, and CPE-1 Extracts. (**E**,**F**) Reducing power assay of COD, COE (**E**), and CPE-1 (**F**) in comparison to AA and BHT, showing dose-dependent antioxidant activity. (**G**) DNA protective assay showing the protective effect of COD, COE, and CPE-1 extracts on plasmid DNA against oxidative damage. The presence of supercoiled and open circular forms indicates DNA integrity. Evaluation of the Antioxidant and Cytotoxic Activities of COD, COE, and CPE-1 Extracts. (**H**–**J**) Cytotoxicity assessment (MTT assay) of COD (**H**), COE (**I**), and CPE-1 (**J**) extracts on RAW264.7 cells across a concentration range (10–1000 µg/mL). Cell viability was calculated as a percentage relative to untreated control (C). Data are presented as mean ± SD. The antioxidant and cytotoxic effects were concentration-dependent, with varying efficacy observed among the extracts.

Previous studies have reported that *Chamaecyparis obtusa* contains bioactive compounds such as terpenoids and flavonoids, which contribute to its cytotoxic and antioxidant properties [13,33]. In the present study, both the ethanolic (COE) and aqueous (COD) extracts of *C. obtusa* exhibited antioxidant activities. As shown in Figure 2A COD and COE demonstrated remarkable DPPH radical-scavenging activity, indicative of their potent hydrogen- and electron-donating capacities, comparable to that of the standard antioxidant BHT. Interestingly, the bioconverted extract CPE-1 Figure 2B exhibited the most effective DPPH scavenging activity among the tested samples.

The broad antioxidant potential of COD and COE was further confirmed by the ABTS radical-scavenging assay Figure 2C, where both extracts exhibited scavenging capacities similar to that of ascorbic acid (AA). CPE-1 also demonstrated comparable ABTS scavenging ability, suggesting the retention of substantial antioxidant activity after bioconversion. Reducing power assays Figure 2E. provided additional evidence of the antioxidant efficacy of these extracts, with COD showing slightly greater ferric-reducing activity than COE, while CPE-1 displayed comparatively lower reducing capacity.

DNA protection assays (Figure 2G) revealed that none of the extracts, including CPE-1, caused detectable DNA damage, as indicated by the preservation of supercoiled and open circular plasmid forms. Furthermore, MTT assays were conducted to assess the cytotoxicity of the extracts (Figure 2H–J). COD, COE, and CPE-1 exhibited low cytotoxicity across the tested concentration range, indicating high biocompatibility. However, CPE-1 displayed a mild dose-dependent cytotoxic effect, suggesting enhanced biological activity after bioconversion with *Phellinus linteus* mycelium.

These results are consistent with previous studies reporting that *P. linteus* bioconversion can enhance the bioactivity of natural compounds by structural modification, leading to increased anticancer or immunomodulatory potential [34,35]. Collectively, these findings confirm that *C. obtusa* extracts possess strong antioxidant potential with minimal cytotoxicity, and that bioconversion (CPE-1) may selectively enhance certain bioactive properties without compromising safety.

### 3.3. Comparative Analysis of the Antibacterial Effects of COE, COD, and CPE-1 on Staphylococcus aureus and Bacillus

The antibacterial activities of COE, COD, and CPE-1 were evaluated against *Staphylococcus aureus* and *Bacillus* spp. using the agar well diffusion method (Table 2). Consistent with previous reports indicating that *Chamaecyparis obtusa* contains terpenoids with antimicrobial potential [33]. All extracts exhibited measurable inhibition zones, although the magnitude of activity varied among the samples.

The ethanolic extract (COE) demonstrated the strongest antibacterial activity, producing the largest inhibition zones (20–24 mm) at concentrations of 25–100 mg/mL. This finding suggests that ethanol efficiently extracts lipophilic antibacterial constituents, such as terpenoids, known to contribute to the antimicrobial properties of *C. obtusa.* The aqueous extract (COD) exhibited moderate inhibition (6–15 mm), indicating that water-soluble antibacterial metabolites are also present.

In contrast, the bioconverted extract (CPE-1) showed comparatively smaller inhibition zones (10–14 mm) across tested concentrations. Although CPE-1 exhibited enhanced antioxidant and anti-inflammatory properties in the present study, its reduced antibacterial activity may be attributed to *Phellinus linteus*-mediated biotransformation. Such bioconversion processes can structurally modify or metabolise antibacterial terpenoids, thereby reducing their abundance while generating metabolites with alternative biological functions, as documented in related studies [36,37,38,39]. These results are consistent with other biological assays performed in this study. While CPE-1 demonstrated superior antioxidant capacity, COE and COD retained more pronounced antibacterial effects with minimal cytotoxicity. Taken together, the findings suggest that *P. linteus* biotransformation selectively modulates the phytochemical composition of *C. obtusa*, shifting the bioactive profile from antibacterial activity toward enhanced antioxidant and inflammation-regulating functions.

### 3.4. Inhibitory Effects of COD, COE, and CPE-1 on LPS-Stimulated Inflammatory Responses in RAW 264.7 Cells

**Figure 3:**
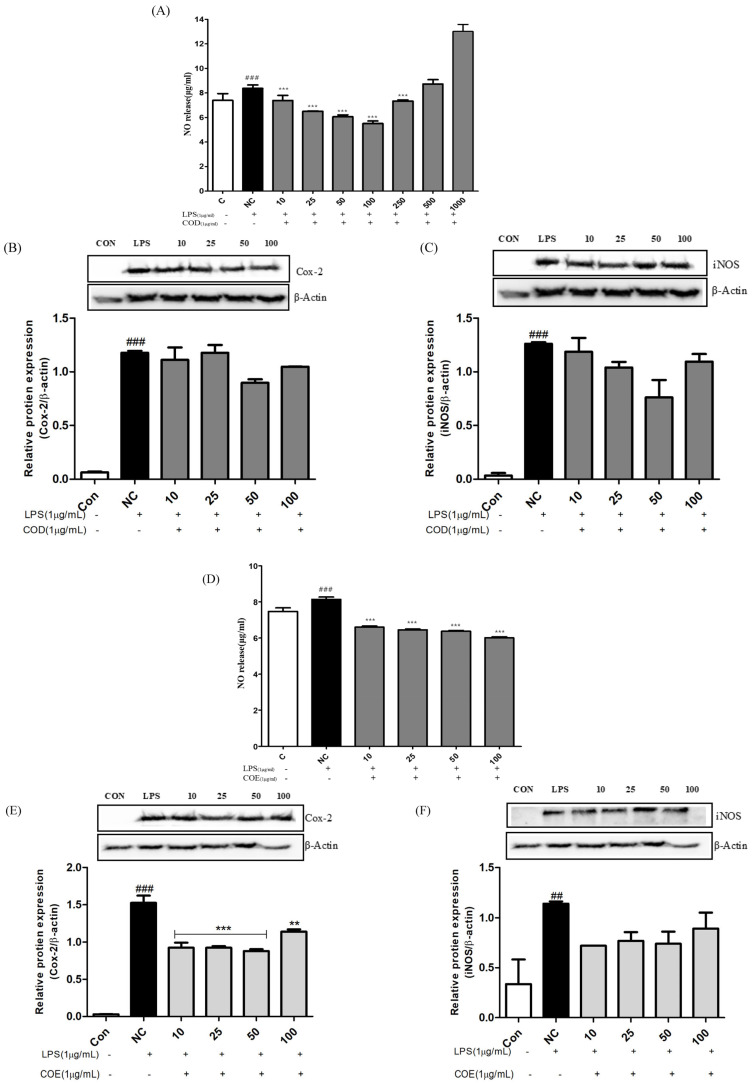

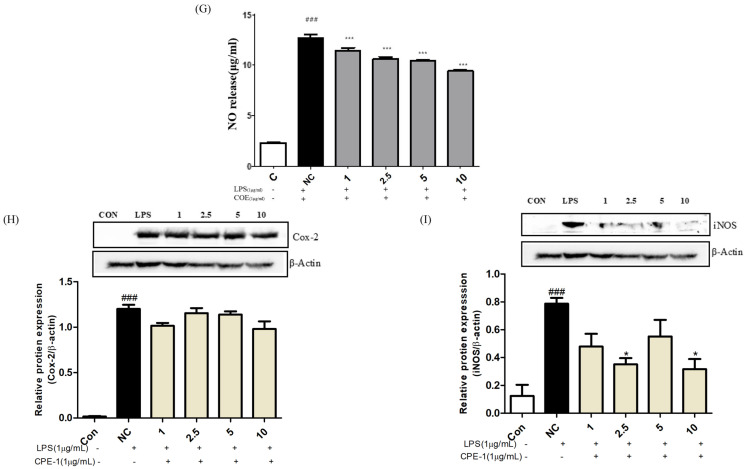
Inhibitory effects of *Chamaecyparis obtusa*-derived samples (COD) on LPS-stimulated inflammatory responses in RAW 264.7 macrophages. (**A**) Nitric oxide (NO) production was measured in culture supernatants after 24 h of treatment with various concentrations of COD in the presence of LPS (1 µg/mL). Western blot analysis of COX-2 and iNOS protein expression in LPS-stimulated RAW 264.7 cells treated with increasing concentrations of COD (**B**,**C**). β-Actin was used as a loading control. Bar graphs represent the relative protein expression normalized to β-actin, expressed as the mean ± SD of three independent experiments. C: untreated control group; NC: LPS-stimulated negative control; treatment groups were co-treated with LPS (1 µg/mL) and varying concentrations of the respective extracts (10–100 µg/mL for COD. Inhibitory effects of *Chamaecyparis obtusa*-derived samples (COE) on LPS-stimulated inflammatory responses in RAW 264.7 macrophages. (**D**) Nitric oxide (NO) production was measured in culture supernatants after 24 h of treatment with various concentrations of COE in the presence of LPS (1 µg/mL). Western blot analysis of COX-2 and iNOS protein expression in LPS-stimulated RAW 264.7 cells treated with increasing concentrations of COE (**E**,**F**). β-Actin was used as a loading control. Bar graphs represent the relative protein expression normalized to β-actin, expressed as the mean ± SD of three independent experiments. C: untreated control group; NC: LPS-stimulated negative control; treatment groups were co-treated with LPS (1 µg/mL) and varying concentrations of the respective extracts (10–100 µg/mL COE. Inhibitory effects of *Chamaecyparis obtusa*-derived samples (CPE-1) on LPS-stimulated inflammatory responses in RAW 264.7 macrophages. (**G**) Nitric oxide (NO) production was measured in culture supernatants after 24 h of treatment with various concentrations of CPE-1 in the presence of LPS (1 µg/mL). Western blot analysis of COX-2 and iNOS protein expression in LPS-stimulated RAW 264.7 cells treated with increasing concentrations of CPE-1 (**H**,**I**). β-Actin was used as a loading control. Bar graphs represent the relative protein expression normalised to β-actin, expressed as the mean ± SD of three independent experiments. C: untreated control group; NC: LPS-stimulated negative control; treatment groups were co-treated with LPS (1 µg/mL) and varying concentrations of the respective extracts (1–5 µg/mL for CPE-1). Data represent mean ± SD (*n* = 3). ## *p* < 0.01, ### *p* < 0.001 vs. control (C); * *p* < 0.05, ** *p* < 0.01, *** *p* < 0.001 vs. LPS-stimulated (NC) group.

The anti-inflammatory effects of *Chamaecyparis obtusa*–derived extracts (COD, COE, and CPE-1) were evaluated using LPS-induced RAW 264.7 macrophages. As shown in Figure 3A,C,G, treatment with all extracts significantly reduced nitric oxide (NO) production in a concentration-dependent manner compared with the LPS-treated control group (### *p* < 0.001 vs. control; *** *p* < 0.001 vs. LPS). Among the three extracts, CPE-1 exhibited the most pronounced inhibitory effect on NO release, suggesting its strong anti-inflammatory potential.

To further elucidate the molecular mechanisms underlying these effects, Western blot analysis was performed to assess the expression of inflammation-related proteins COX-2 and iNOS. Treatment with COD, COE, and CPE-1 markedly reduced the LPS-induced upregulation of both COX-2 and iNOS compared with the untreated control Figure 3B,C,E,F,H,I Among the extracts, CPE-1 demonstrated the most substantial downregulation of these proinflammatory markers, indicating superior efficacy in suppressing inflammatory signalling in macrophages. These results are consistent with previous studies reporting the anti-inflammatory potential of *C. obtusa*–derived bioactive constituents [17]. Interestingly, although CPE-1 exhibited slightly higher antioxidant and antibacterial activity in prior assays, it showed comparable inhibitory effects on proinflammatory mediators. This finding suggests that bioconversion using *Phellinus linteus* may modify or enhance the bioactive profile of *C. obtusa*, thereby improving its anti-inflammatory capacity [2,20].

Overall, these findings demonstrate that *C. obtusa* extracts, particularly CPE-1, exert potent inhibitory effects on LPS-induced inflammatory responses in RAW 264.7 macrophages.

### 3.5. Suppression of LPS-Induced Inflammatory Responses by COD, COE, and CPE-1 via MAPK Pathway Modulation in RAW 264.7 Macrophages

**Figure 4:**
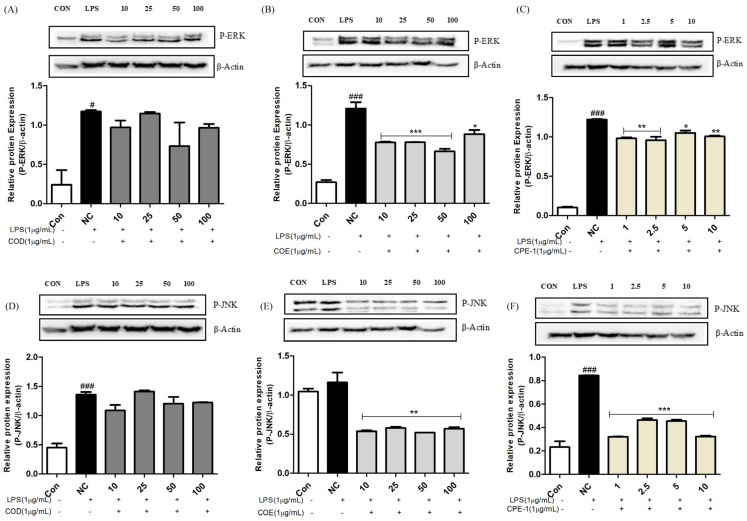
Suppression of LPS-induced inflammatory responses by *Chamaecyparis obtusa*-derived samples (COD, COE, and CPE-1) via MAPK pathway modulation in RAW 264.7 macrophages. (**A**–**C**) Western blot analysis of phosphorylated ERK (p-ERK) and corresponding quantitative densitometry in LPS-stimulated RAW 264.7 cells treated with various concentrations of COD (**A**), COE (**B**), or CPE-1 (**C**). Suppression of LPS-induced inflammatory responses by *Chamaecyparis obtusa*-derived samples (COD, COE, and CPE-1) via MAPK pathway modulation in RAW 264.7 macrophages. (**D**–**F**) Western blot analysis of phosphorylated JNK (p-JNK) and corresponding densitometric analysis in LPS-stimulated cells treated with increasing concentrations of COD (**D**), COE (**E**), or CPE-1 (**F**). β-Actin was used as an internal loading control. Bar graphs represent the relative protein expression normalised to β-actin, expressed as the mean ± SD of three independent experiments. ### *p* < 0.001, # *p* < 0.05 versus control (Con); *** *p* < 0.001, ** *p* < 0.01, * *p* < 0.05 versus LPS-treated group (NC). C: control group (untreated); NC: negative control (LPS-stimulated without extract treatment); treated groups received LPS and indicated doses of extract.

The MAPK signalling cascade is a key mediator of inflammatory responses in macrophages exposed to LPS stimulation. To further elucidate the molecular mechanism underlying the anti-inflammatory effects of *Chamaecyparis obtusa*–derived extracts, the effects of COD, COE, and CPE-1 on MAPK pathway activation were examined in LPS-stimulated RAW 264.7 cells. As shown in Figure 4, LPS treatment markedly enhanced the phosphorylation of ERK and JNK, confirming robust MAPK activation under inflammatory conditions. In contrast, treatment with all three extracts significantly attenuated this phosphorylation in a concentration-dependent manner (*** *p* < 0.001 vs. LPS). Specifically, CPE-1 exhibited the most pronounced inhibitory effect on both ERK (Figure 4A–C) and JNK (Figure 4D–F) phosphorylation, while COD and COE showed moderate but dose-dependent suppression of MAPK activation.

These findings align well with the anti-inflammatory effects described in Figure 3, where COD, COE, and CPE-1 effectively suppressed NO production and downregulated COX-2 and iNOS expression. The combined data suggest that inhibition of the MAPK signalling cascade contributes significantly to the anti-inflammatory mechanisms of *C. obtusa* extracts.

Moreover, the inhibitory activities of COD and COE on ERK and JNK phosphorylation correspond with their previously demonstrated antioxidant capacities, including reducing power and radical-scavenging activity. Notably, although CPE-1 exhibited relatively higher antioxidant and antibacterial activities, it maintained and in some cases enhanced its MAPK-inhibitory potential. This observation implies that *Phellinus linteus*–mediated bioconversion may alter or enrich the bioactive chemical composition of *C. obtusa*, thereby enhancing specific anti-inflammatory functions. Such effects are consistent with earlier reports that fungal bioconversion can potentiate the immunomodulatory and cytotoxic activities of natural products [13,18,21].

Collectively, these results demonstrate that *C. obtusa* extracts, particularly the bioconverted extract CPE-1, effectively suppress LPS-induced MAPK signalling in macrophages.

### 3.6. Characterisation of Bioactive Constituents from COE and CPE-1 Using Spectroscopic and Chromatographic Techniques

**Figure 5:**
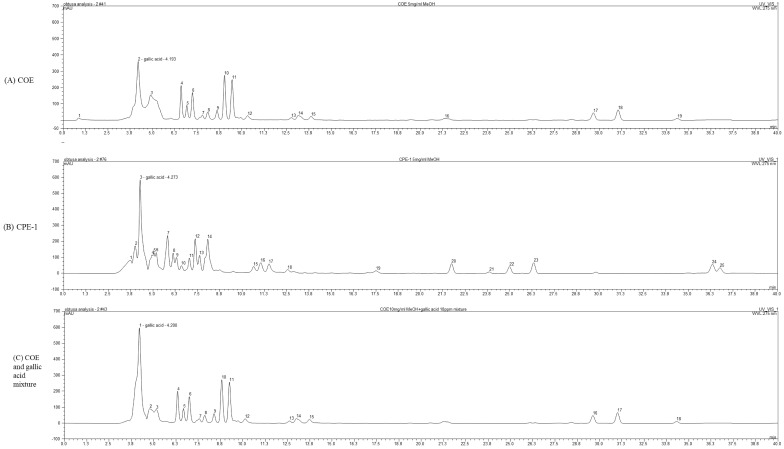
The chromatographic peaks around 4.19–4.27 min in all three samples—(**A**) COE, (**B**) CPE-1—correspond to gallic acid, and (**C**) COE + gallic acid mixture, based on retention time alignment: (**A**) COE alone shows a peak at ~4.193 min. (**B**) COE + gallic acid mixture shows gallic acid at ~4.200 min. (**C**) CPE-1 (bioconverted extract) shows a peak at ~4.273 min.

The HPLC chromatograms of the ethanol extract (COE), the bioconverted extract (CPE-1), and the gallic acid combination are presented in Figure 5. A distinct peak corresponding to gallic acid was observed in all samples, appearing at retention times between 4.19 and 4.27 min. Specifically, gallic acid was detected at 4.193 min in COE, 4.200 min in the gallic acid mixture, and 4.273 min in CPE-1. This alignment confirms that gallic acid, a key phenolic constituent, is preserved during bioconversion by *Phellinus linteus*.

Although both COE and CPE-1 retained gallic acid, their overall chromatographic patterns exhibited notable differences, suggesting that significant phytochemical modifications occurred following bioconversion. Such compositional changes are likely responsible for the altered biological activities observed in the bioassays.

Consistent with these chemical findings, both COE and CPE-1 demonstrated marked anti-inflammatory activity by downregulating COX-2 and iNOS expression, suppressing nitric oxide production, and inhibiting MAPK pathway activation (ERK and JNK phosphorylation). These effects can be attributed to the bioactive compounds that were either maintained or newly generated during the bioconversion process. Furthermore, the presence of gallic acid and other bioconversion-derived metabolites may contribute to the enhanced antibacterial and cytotoxic properties of *C. obtusa* extracts, thereby improving their cell-targeting efficacy and overall biofunctional potential.

### 3.7. Evaluation of CPE-1 Intracellular Reactive Oxygen Species in RAW 264.7 Cells Using Flow Cytometry

**Figure 6:**
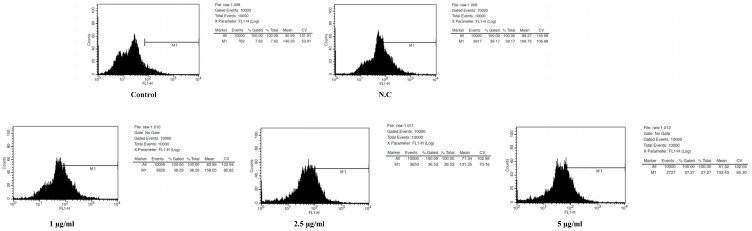
Flow Cytometry Analysis Highlights: N.C (Negative Control) shows the highest ROS levels (Mean FL1-H = 168.75). CPE-1-treated cells show decreased ROS levels at all tested concentrations: 1 µg/mL: Mean = 158.05, 2.5 µg/mL: Mean = 131.35, 5 µg/mL: Mean = 133.43 and Control (C): Mean = 140.33.

The impact of CPE-1 on ROS generation in LPS-stimulated RAW 264.7 cells is shown in Figure 6. As seen by a shift in fluorescence intensity, LPS significantly raised ROS levels, whereas CPE-1 administration decreased this impact in a concentration-dependent manner (1–5 µg/mL). These findings imply that CPE-1 successfully reduces oxidative stress. The decrease in ROS is in line with *Chamaecyparis obtusa*’s well-known antioxidant qualities, which include the presence of phenolic substances like gallic acid. Furthermore, bioconversion with the fungus *Phellinus linteus*, which has anti-inflammatory and antioxidant properties, might increase this activity. Extracts from *Phellinus linteus* have been shown in earlier research to lower ROS levels and inflammatory reactions.

Since inflammation is mainly triggered by oxidative stress, CPE-1’s capacity to limit ROS supports its anti-inflammatory properties, which are further supported by related research showing that it inhibits the MAPK pathway and proinflammatory indicators. Oxidative stress regulation may potentially be partly responsible for the cytotoxic effects of *Chamaecyparis obtusa* that have been noted in previous investigations [2,40]. Therefore, CPE-1’s ability to reduce ROS may further promote its multifunctional therapeutic potential by contributing to its overall bioactivity profile.

## 4. Discussion

The present study examined the biological activities of ethanolic (COE), aqueous (COD), and bioconverted (CPE-1; *Phellinus linteus* mycelium–treated) extracts of *Chamaecyparis obtusa*. Among them, CPE-1 exhibited the most potent antioxidant and anti-inflammatory activities, while showing comparatively smaller antibacterial inhibition zones (10–14 mm). These results suggest that fungal biotransformation significantly enhanced the overall bioactive potential of *C. obtusa*. This indicates that *P. linteus*–mediated conversion altered the phytochemical profile, possibly producing metabolites with improved biological efficacy.

Both COE and COD demonstrated notable antioxidant properties, consistent with previous reports attributing such effects to flavonoids and terpenoids in *C. obtusa* [33]. However, CPE-1 showed markedly higher radical-scavenging activities in DPPH and ABTS assays, comparable to standard antioxidants such as ascorbic acid and BHT. Although COD showed slightly greater ferric-reducing capacity, CPE-1’s superior direct scavenging efficiency indicates a stronger overall antioxidant potential. Moreover, DNA protection assays confirmed that its cytotoxic activity was selective and did not compromise genomic integrity.

In LPS-stimulated RAW 264.7 macrophages, all extracts significantly inhibited nitric oxide (NO) production and suppressed COX-2 and iNOS expression, with CPE-1 showing the greatest inhibition. These effects corresponded with reduced phosphorylation of ERK and JNK, implicating modulation of the MAPK pathway—a key regulator of inflammatory and oxidative responses [30,31]. The inhibition of ERK and JNK phosphorylation by CPE-1 suggests that fungal biotransformation enhanced the anti-inflammatory capacity of *C. obtusa* through attenuation of MAPK-mediated signalling cascades.

In addition, CPE-1 markedly reduced intracellular ROS accumulation, linking its antioxidant and anti-inflammatory effects. This dual activity may involve activation of the Nrf2/HO-1 pathway, which governs cellular defence against oxidative stress by promoting the expression of detoxifying and antioxidant enzymes [41]. Activation of Nrf2 not only enhances ROS scavenging but also negatively regulates NF-κB signalling, thereby reducing the transcription of proinflammatory genes such as COX-2 and iNOS [42]. Such cross-regulation between Nrf2 and NF-κB pathways is well recognised as a central mechanism by which phenolic compounds and fungal-derived metabolites exert their cytoprotective effects.

HPLC profiling confirmed gallic acid as a major phenolic present in both COE and CPE-1. Gallic acid has been widely reported to exert potent antioxidant and anti-inflammatory activities by scavenging ROS, suppressing NF-κB activation, and reducing the expression of inflammatory enzymes [43,44]. Quercetin and quercetin-derived metabolites, also identified in *C. obtusa* and known for strong antioxidant and anti-inflammatory functions, have been shown to modulate MAPK and Nrf2 pathways, suppress NO production, and inhibit inflammatory cytokines [45,46]. The presence of these compounds likely contributed to the observed bioactivities of the extracts.

Nevertheless, the superior efficacy of CPE-1 implies that fungal biotransformation via *P. linteus* generated additional bioactive metabolites or enhanced the bioavailability of existing compounds. Previous studies have demonstrated that fungal fermentation particularly by Basidiomycetes can increase total phenolic content, produce novel phenolic derivatives, and substantially elevate antioxidant capacity [47,48]. Similar enhancements in bioactivity following microbial transformation have been reported for other plant materials, supporting the role of fungi in producing novel or more potent phenolic derivatives [36,49,50].

Overall, the present findings indicate that *P. linteus*–mediated bioconversion of *C. obtusa* significantly improved its antioxidant and anti-inflammatory potential, primarily through modulation of redox-sensitive signalling pathways such as Nrf2/HO-1, NF-κB, and MAPK. These results highlight the potential of fungal biotransformation as an efficient strategy for producing multifunctional natural products with pharmaceutical and functional food applications.

## 5. Conclusions

This study demonstrates that bioconversion of *Chamaecyparis obtusa* extracts using *Phellinus linteus* mycelium significantly enhances their biological efficacy by transforming native plant precursors into more potent bioactive metabolites. Among the tested samples, the bioconverted extract (CPE-1) exhibited superior antioxidant and anti-inflammatory activities compared with the ethanolic (COE) and aqueous (COD) extracts, although it showed relatively smaller antibacterial inhibition zones. Notably, CPE-1 presented strong radical-scavenging activity comparable to standard antioxidants such as ascorbic acid and BHT, while maintaining genomic stability.

At the cellular level, CPE-1 significantly inhibited nitric oxide (NO) production and reduced the expression of key inflammatory mediators, including COX-2 and iNOS, alongside suppressing MAPK signalling through ERK and JNK phosphorylation. These findings indicate that the enhanced activity of CPE-1 is attributable to fungal-mediated modification of its phytochemical composition, generating new or more active secondary metabolites while preserving stable compounds such as gallic acid.

This study highlights a notable biotechnological advancement in the valorisation of *C. obtusa* through *P. linteus*-assisted biotransformation. Unlike conventional extraction or fermentation methods, this approach leverages the enzymatic machinery of a medicinal fungus to enhance phenolic bioavailability and biological potency, an innovation that aligns with recent reports on the fungal-mediated enhancement of plant metabolites. The results collectively demonstrate the potential of fungal biotransformation as a sustainable platform for developing multifunctional bioactive materials applicable to functional foods, nutraceuticals, and therapeutic formulations.

### Limitations and Future Perspectives

While this study provides strong evidence that *Phellinus linteus*-mediated bioconversion enhances the biological efficacy of *Chamaecyparis obtusa* extracts, several limitations should be acknowledged. First, the current experimental design did not include a mycelium-only control group, which would allow a clear distinction between the intrinsic bioactivities of *P. linteus* mycelium and the synergistic or transformation-related effects produced during the bioconversion process. Incorporating such a control in future studies will help fully isolate and quantify the contributions of the fungal substrate itself versus the metabolites generated through biotransformation.

Additionally, the phytochemical characterisation in this study was limited to HPLC-based quantification of known phenolic and flavonoid compounds. Although this approach effectively demonstrated enhanced biochemical activity and confirmed the retention of key marker compounds such as gallic acid, it did not permit the comprehensive identification of newly generated or structurally modified metabolites produced during the bioconversion process.

Future research will therefore employ advanced analytical tools such as LC–MS/MS and NMR spectroscopy to confirm, identify, and structurally elucidate the novel metabolites formed during fungal transformation. These analyses will provide deeper insight into the specific metabolic pathways facilitated by *P. linteus* and how these pathways relate to the improved antioxidant, antimicrobial, and anti-inflammatory properties observed in CPE-1.

Moreover, subsequent studies will investigate the bioavailability, pharmacokinetics, and in vivo efficacy of these bioconverted metabolites to better establish their potential applications in functional food, nutraceutical, and therapeutic formulations. Integrating these approaches will advance our understanding of fungal biotransformation and support the development of scientifically validated bioactive products derived from *C. obtusa*.

## Figures and Tables

**Table 1:** Total phenolic, Flavonoid assay, All data are expressed as mean ± standard deviation (*n* = 3).

Extract	Total Phenolic (µg GAE †/mg of Extract)	Total Flavonoid (µg CE ††/mg of Extract)
COD	366.246 ± 8.76 µg	26.751 ± 1.31 µg
COE	177.1692 ± 7.899 µg	109.6914 ± 2.823 µg
CPE-1	62.90404 ± 3.999907 µg	137.8148 ± 3.591972 µg

† GAE: gallic acid equivalent, †† CE: catechin equivalent; COD: *Chamaecyparis obtusa* DW extract; COE: *Chamaecyparis obtusa* EtOH extract; CPE-1: *Chamaecyparis obtusa* EtOH extract bioconverted with *Phellinus linteus* mycelium.

**Table 2:** Antibacterial activity of COE, COD Extract, CPE-1 in *Staphylococcus aureus* and *Bacillus*.

Extract	COD		COE		CPE-1	
	Concentration (mg/mL)	Size	Concentration (mg/mL)	Size	Concentration (mg/mL)	Size
*Staphylococcus aureus*	500 mg/mL	15 mm	100 mg/mL	20 mm	100 mg/mL	12 mm
	250 mg/mL	13 mm	50 mg/mL	21 mm	10 mg/mL	13 mm
	100 mg/mL	10 mm	25 mg/mL	21 mm	1 mg/mL	10 mm
	-	-	10 mg/mL	16 mm	-	-
*Bacillus*	500 mg/mL	10 mm	100 mg/mL	20 mm	100 mg/mL	14 mm
	250 mg/mL	8 mm	50 mg/mL	24 mm	10 mg/mL	13 mm
	100 mg/mL	6 mm	25 mg/mL	24 mm	1 mg/mL	11 mm
	-		10 mg/mL		-	

## Data Availability

The original contributions presented in this study are included in the article. Further inquiries can be directed to the corresponding author.

## Abbreviations

The following abbreviations are used in this manuscript:

COD	*Chamaecyparis obtusa* DW extract
COE	*Chamaecyparis obtusa* EtOH extract
CPE-1	*Chamaecyparis obtusa* EtOH extract bioconverted with *Phellinus linteus* mycelium
iNOS	Inducible nitric oxide synthase
COX-2	Cyclooxygenase-2
MAPK	Mitogen-activated protein kinase
Erk	Extracellular Signal-Regulated Kinase
JNK	c-Jun N-terminal kinase
NO	Nitric oxide
FBS	Foetal bovine serum
DMEM	High-glucose Dulbecco’s modified Eagle medium
